# Making Health Services Adolescent-Friendly in Northeastern Peninsular Malaysia: A Mixed-Methods Study

**DOI:** 10.3390/ijerph17041341

**Published:** 2020-02-19

**Authors:** Hafizuddin Awang, Azriani Ab Rahman, Surianti Sukeri, Noran Hashim, Nik Rubiah Nik Abdul Rashid

**Affiliations:** 1 Department of Community Medicine, School of Medical Sciences, Universiti Sains Malaysia, Kubang Kerian 16150, Kelantan, Malaysia; hafizuddinawang@yahoo.com (H.A.); azriani@usm.my (A.A.R.); 2 Maternal and Child Health Unit, Kelantan State Health Department, Kota Bharu 15590, Kelantan, Malaysia; drnoran@moh.gov.my; 3 Adolescent Health Sector, Family Health Development Division, Ministry of Health, Parcel E, Federal Government Administration Centre, Putrajaya 62590, Malaysia; rubiah@moh.gov.my

**Keywords:** facilitating factors, adolescent-friendly health services, mixed-methods study, Malaysia

## Abstract

A mixed-methods study was conducted in a Malaysian state beginning with a cross-sectional quantitative study to determine the relationship between clinic characteristics and clinic score of adolescent-friendliness. Subsequently, perceptions of healthcare providers on the facilitating factors for the provision of adolescent-friendly health services were explored qualitatively to support the quantitative findings. Availability of trained healthcare providers, adequate privacy, dedicated adolescent health services team, and adolescent health promotional activities were the clinic characteristics that significantly (*p* < 0.05) related with clinics’ scores of adolescent-friendliness. The facilitating factors required for adolescent-friendly health services were (1) healthcare providers’ commitment and prioritization towards adolescent-friendly health services; (2) organizational supports; (3) appropriate clinic settings; and (4) external supports for adolescent health promotional activities. The qualitative findings reaffirmed those of the quantitative study on the significant clinic characteristics required for adolescent-friendly health services. This study provides valuable insight for the Ministry of Health to elicit the required facilitating factors to further improve the quality of adolescent health services in Malaysia.

## 1. Introduction

Adolescents are defined as a group of population between the ages of 10 and 19 years old [1]. In Malaysia, adolescence is further subcategorized into early adolescence (10–14 years), middle adolescence (15–17 years), and late adolescence (18–19 years) [2]. There are about 1.2 billion adolescents in the world today, making up 16% of the world’s population [3]. Southeast Asian region, of which Malaysia is part of it, consists of 213 million adolescents, and as for Malaysia, the total number of adolescents is 5.5 million or around 18% of its 31 million population [2,4]. Undeniably, the adolescents group is an important category of a country’s demography and is in pressing need of attention, as adolescents are both our present and future leaders, catalyzing economic, social, and cultural development [4].

Given the significant proportion of population and socio-economic values of adolescents in Malaysia, the Ministry of Health introduced the adolescent health services in 1996 to develop and strengthen health services for young people, in line with the objectives set out in the National Youth Health Policy of Malaysia [5]. These services include physical health, healthy eating, mental health, behavioral health, and sexual and reproductive health; which are provided in all government primary healthcare facilities throughout Malaysia [6]. However, a recent report from the Ministry of Health, Malaysia stated that the current adolescent health services in Malaysia are not youth-friendly enough. These health services are deemed less visible than other health services and are often underutilized by adolescent clients [7]. A cross-sectional survey done in one of the Malaysian states among 680 adolescents reported very low utilization of the adolescent sexual and reproductive health services, as only 6.9% of them had ever visited primary healthcare facilities [8]. According to previous studies among Malaysian adolescents, the main barriers to the utilization of adolescent health services were lack of information on these services in the primary healthcare facilities, negative attitude of healthcare providers, lack of privacy, and fear of family and friends knowing about their health issues [9,10]. Furthermore, Ghafari, Shamsuddin, and Amiri [9] reported that Malaysian adolescents insisted on privacy and confidentiality during visit to clinics, favoring adolescent-friendly health facilities that bear no stigma towards their health problems, with highly trained support staff to treat them with respect.

Failure to fulfill the needs of adolescent clients optimally may have serious implications towards their health. Previous Malaysian studies reported multiple morbidities and mortalities among adolescents resulting from unintentional injuries, risky behaviors, and infectious and non-communicable diseases [11,12,13,14,15]. Worse, Malaysian adolescents may resort to cigarette smoking, alcohol consumption, recreational drugs use, and committing suicide as solutions to their personal problems since they are unaware of the adolescent health services targeting these issues [10,16]. Therefore, adolescents are at risk of premature morbidity and mortality if no preventive measures are taken. Adolescents should enjoy the highest attainable standards of health with a supportive environment, particularly on health services [17]. Thus, it is imperative for adolescent-friendly health services to be equitable, accessible, acceptable, appropriate, and effective following the World Health Organization (WHO)-defined dimensions of quality health services to adolescents [18].

To ensure adolescent health services meet the health needs of adolescents and comply with the WHO-defined dimensions of quality health services to adolescents, the Ministry of Health, Malaysia introduced the national best practices for adolescent-friendly health services in Malaysia in 2018 [7,19]. The outlined criteria, adapted from the WHO characteristics for adolescent-friendly health services [18], guided the restructuring of primary healthcare facilities in Malaysia to become more adolescent-friendly. Our pilot study on these Malaysian best practices for adolescent-friendly health services revealed only 35.3% of health clinics in Kelantan, a northeastern region of Peninsular Malaysia, were accredited as adolescent-friendly health facilities [19]. Evidently the provision of adolescent-friendly health services in Malaysia remains suboptimal and need further improvement. To assist policymakers in strengthening the current adolescent health services, input from the perspective of healthcare providers on the facilitating factors for adolescent-friendly health services provision is much needed.

To the best of our knowledge, no study was done in Malaysia to explore the requirements of adolescent-friendly health services from the perspective of Malaysian healthcare providers. Existing Malaysian studies on facilitating factors of adolescent-friendly health services were investigated from the perspective of adolescents and they were more focused on the adolescent mental health and sexual and reproductive health services only [8,9,10]. Additional exploration from the perspective of healthcare providers would complement the adolescents’ findings and may be useful towards improving adolescent-friendly health services in Malaysia. Elsewhere, qualitative exploration among healthcare providers reported that unique clinic settings, support from various stakeholders, and commitments of healthcare providers were among the prerequisites for the provision of adolescent-friendly health services in health facilities [20,21,22]. Due to the scarcity of the local information on the ideal settings for providing such services, we carried out this mixed-method study to fill the research gaps. Therefore, this study aimed to (1) determine the relationship between clinic characteristics and clinic score of adolescent-friendliness; and (2) explore the perception of healthcare providers on the facilitating factors towards providing adolescent-friendly health services in a Malaysian setting.

## 2. Materials and Methods

### 2.1. Study Design

To answer the study questions, researchers used both quantitative and qualitative methods within a single research project. This type of study design allowed us to explain the findings obtained from the quantitative part (testing on the relationship between clinic characteristics and clinic assessment scoring of adolescent-friendliness) through qualitative exploration among healthcare providers on the perception and facilitating factors in providing adolescent-friendly health services given the limited availability of validated and reliable questionnaire to be used for this purpose [23]. This type of study design is best known as the sequential explanatory type of mixed-methods approach, using triangulation design to gain complementary data on the same topic and to best understand about adolescent-friendly health services [24].

### 2.2. Quantitative Phase

The first phase of the study began with the aim to determine the relationship between clinic characteristics and clinic score of adolescent-friendliness. This cross-sectional study was conducted over a four-month period from November 2018 until February 2019 in Kelantan, a Malay ethnic-dominant state located in the northeastern region of Peninsular Malaysia. Statewide assessment of 85 health clinics was done in our previous study to determine the proportion of adolescent-friendly clinics in Kelantan [19]. In our previous study, data were collected through clinics assessment by trained enumerators for compliance with the twelve criteria outlined in national best practices for adolescent-friendly health services. An audit checklist adapted from WHO characteristics of adolescent-friendly health services suited to the Malaysian healthcare setting was used as the standard tool in the assessment of clinics [7]. Based on assessment, clinics with a score of 80% and above were accredited as adolescent-friendly clinics. Clinics that scored less than 80% were regarded as conventional clinics [7]. Methodological details on the statewide clinic assessment, study criteria, and its findings can be found in our previous study [19]. Besides assessing clinics for their adolescent-friendliness scoring, another important aspect of the study was to assess for their characteristics in order to relate them with the clinic assessment score. The study inclusion criteria were universal-, intermediate-, and advanced-type clinics; while rural and community clinics were excluded from this study as they do not offer daily adolescent health services [25].

To determine the relationship between clinic characteristics and clinic score of adolescent-friendliness, the required sample size was calculated using G*Power calculation for multiple linear regression test [26]. The estimated sample needed was 81 clinics using effect size f^2^ of 0.1, 5% type 1 error, and 80% power. Out of the 281 clinics in Kelantan, only 85 clinics fulfilled the study criteria while another 196 health facilities were rural and community clinics. Therefore, we included all 85 eligible clinics in the study [25].

Data on the characteristics of clinics were collected through clinics’ assessment by trained enumerators. Clinic pro forma containing information on clinic name and its characteristics were obtained. The eight clinic characteristics were (1) number of healthcare providers (medical officers, assistant medical officers, and nurses); (2) daily patients’ attendance; (3) type of clinics (advanced, intermediate, or universal); (4) availability of family medicine specialists at clinic; (5) availability of highly trained healthcare providers in adolescent health management; (6) availability of private room or space for counseling session; (7) availability of dedicated team in charge of adolescent health services; and (8) availability of promotional activities to promote adolescent health services to adolescents and community.

Universal-, intermediate-, and advanced-type clinics were defined in accordance with their daily patients’ attendance and extent of services offered at the clinics. Universal clinics have daily patients’ attendance of less than 50 and are equipped with paramedics and nurses; while intermediate clinics usually have daily attendance between 50 to 150 patients and are equipped with medical officers. Advanced clinics are the most advantaged type as they are equipped with medical officers, family medicine specialists, multidisciplinary team, medical imaging modalities, and they have an average attendance of at least 300 patients per day [25]. For our study’s operational definitions, availability of highly trained healthcare providers in adolescent health management was defined as clinics equipped with healthcare providers who have been trained in all of the Ministry of Health’s adolescent health modules (Guidelines for Adolescent Health Services Implementation in Primary Healthcare Level; Guidelines on Adolescent Sexual and Reproductive Health Management; or Engaging The Adolescents Module Using HEADSS (Home, Education, Activities, Drug use and abuse, Sexual behavior, Suicidality and depression) Framework). Private room or space indicates a convenient location in clinic which can be regarded as a safe environment for adolescents and offering privacy to them [27]. Dedicated team in charge of adolescent health services refers to a team of healthcare providers at the clinic who are technically competent in adolescent-specific areas and can devote adequate time to manage adolescent clients [27]. Meanwhile, promotional activities refer to any adolescent health promotional activities done by the clinics either internally (such as giving out information, education, and communication materials on adolescent health issues and services to clients) or at community level (such as organizing road tours or seminars on adolescent health issues and services) with the aim of creating awareness among population about the availability of adolescent health services at clinics and increasing adolescent attendance to clinics [28].

Data were analyzed using inferential statistics using SPSS Statistics (IBM Corp. Released 2013. IBM SPSS Statistics for Windows, Version 22.0. IBM Corp., Armonk, NY, USA). Simple and multiple linear regression were used for data analysis to determine the relationship between clinic characteristics suitable for adolescent-friendly health services and clinic assessment score of adolescent-friendliness level. A *p* < 0.05 was taken to indicate statistical significance.

### 2.3. Qualitative Phase

This qualitative phase of the study was carried out from February 2019 until April 2019 to explore the perception of healthcare providers on the facilitating factors towards providing adolescent-friendly health services in Kelantan. This qualitative phase helped to explain healthcare providers’ understanding on the ideal settings and facilitating factors on the provision of adolescent-friendly health services at primary healthcare facilities in Kelantan. A descriptive, qualitative, phenomenological design was used, based on the Giorgi method as the method of inquiry [29]. The goal was to delve into the perceptions of individuals with extensive knowledge and experience in providing the current adolescent healthcare services.

For this qualitative phase, in-depth interviews of healthcare providers were conducted by the first author, who is a male public health medical officer (MD, MPH), and supervised by the third author, who is a female expert (PhD) in qualitative study. Both interviewers were trained adequately at various qualitative study workshops prior to the interview sessions. The study respondents were healthcare providers from multilevel categories inclusive of public health physicians, family medicine specialists, medical officers, assistant medical officers, and nurses whom fulfilled the study criteria. Purposive sampling method was applied. The inclusion criteria were consented healthcare providers who work in either adolescent-friendly or conventional clinics previously involved in the quantitative phase of the study. Individuals unable to understand Malay or English language were excluded from the study. Statistically significant quantitative results that required further explanation were identified, and consented healthcare providers were invited for interview through telephone and e-mail. Sampling continued until saturation of data, which occurred during the twenty-third interview.

All 23 in-depth interviews were carried out following a semistructured script that we developed in accordance with the quantitative phase in order to modulate the interviews. Prior to interviews, the semistructured script was pilot tested among public health physicians and medical officers of Community Medicine Department, Universiti Sains, Malaysia. The interview sessions were carried out in Malay or English language, depending on the preference of the participants. The duration of interviews varied from 45 to 60 min, depending on the interviewees’ desire to continue and present their complete ideas. Every healthcare provider participated in the study voluntarily, after signing an informed consent form in accordance with the Declaration of Helsinki. The aims of the study were explained to them, and all participants were assured that their anonymity would be protected. The respondents were identified with codes to ensure their anonymity, identifying each interview with the letter P (for Public Health Physician), F (for Family Medicine Specialist), M (for Medical Officer), A (for Assistant Medical Officer) and N (for Nurse), followed by a sequential number for each of job category.

To trigger respondents to narrate their experiences on adolescent-friendly health services, all interviews began with the question, “in your opinion what are the factors that may facilitate your clinic in providing adolescent-friendly health services?” Attempts were made to conduct interview sessions at their respective workplaces with the least interference from their daily activities. The interviews were audio-recorded and transcribed verbatim. Field notes were jotted down after each interview by the researcher. Participants were given honorarium at the end of the interview session.

The transcribed data were analyzed using thematic analysis in accordance with Braun and Clarke [30], in which we familiarized ourselves with the data to generate initial codes. Subsequently, we searched for themes and reviewed them before we defined and named the specific themes. To ensure rigor of data, the following steps were employed: member check, interview supervision by qualitative expert, prolonged engagement and allocation of adequate time. For evaluation and transferability, samples were recruited with maximum variations from different job categories, type of clinics (adolescent-friendly and conventional clinics), and level of education.

### 2.4. Ethical Considerations

Ethics approval was obtained from the Medical Review and Ethical Committee from National Institute of Health, Ministry of Health, Malaysia (NMRR-18-2838-44398 (IIR)) and Research and Ethics Committee, Universiti Sains, Malaysia (USM/JEPeM/18100582).

## 3. Results

### 3.1. Quantitative Phase

A total of 85 health clinics were assessed for their assessment scores on adolescent-friendliness level and its relationship with the clinic characteristics. Clinic assessment scores on adolescent-friendly level were translated into the proportion of adolescent-friendly clinics in Kelantan, as reported in our previous study [19]. Our previous study reported the proportion of clinics accredited as adolescent-friendly clinics in Kelantan was 35.3% (95% confidence interval (CI): 0.25, 0.46) or 30 clinics out of total 85 clinics. In multiple linear regression analysis, four characteristics of clinic showed a significant relationship with clinic assessment score on adolescent-friendliness level, which are the availability of highly-trained healthcare providers in adolescent health management; availability of private room or space for counseling session; availability of dedicated team in charge of adolescent health services; and availability promotional activities to promote adolescent health services to adolescents and community. Clinics with these characteristics showed a significantly higher level of adolescent-friendliness. The relationship between clinic characteristics suitable for adolescent-friendly health services and clinic assessment score on adolescent-friendliness is summarized in Table 1.

### 3.2. Qualitative Phase

A total of 23 healthcare providers aged between 31 and 59 years old participated in this phase. Attributes of the study participants are presented in Table 2.

Transcripts of healthcare providers’ perception on the facilitating factors for adolescent-friendly health services generated nine categories and eventually, four themes, which included (1) healthcare providers’ commitment and prioritization towards adolescent-friendly health services; (2) organizational supports to advocate adolescent-friendly health services; (3) appropriate clinic settings for adolescent-friendly health services; and (4) external supports for adolescent health promotional activities (Table 3).

### 3.3. Theme 1: Commitment and Priority from Healthcare Providers’ Perspective towards Adolescent-Friendly Health Services

One of the important themes derived from this study was the commitment and prioritization by healthcare providers’ towards adolescent-friendly health services. These were regarded as key elements to ensure successful implementation of adolescent-friendly health services.

#### 3.3.1. Self-Commitment of Healthcare Providers to Provide Adolescent-Friendly Health Services

All 23 respondents agreed firm commitment from healthcare providers brings forth the mindset and behavior that supports successful implementation of adolescent-friendly health services. Self-commitment gives healthcare providers the drive and will to deliver adolescent-friendly health services successfully, regardless of the number of staff and burden of workload at clinics. A 59-year-old female public health physician stated the following:

“For me, it (the success of adolescent-friendly health services) really depends on staff commitment. The number of staff and patient attendance in a clinic do not influence much. In doing something, we must have passion. If FMS (family medicine specialists) and MO (medical officers) were the only ones interested in the program (adolescent-friendly health services), but not other healthcare staff, these health services definitely cannot succeed.”

(P2)

With great commitment from staff, the majority of respondents believed that adolescent-friendly health services can be implemented at any type of clinics, regardless of whether it is universal, intermediate, or advanced. For instance, a 46-year-old female family medicine specialist made the following comment:

“The success of adolescent-friendly clinic does not really depend on the type of clinics. If our staffs are very committed and passionate about adolescent health services, they surely can implement it in any clinic settings.”

(F2)

#### 3.3.2. Prioritization by Top Managers towards the Implementation of Adolescent-Friendly Health Services at Ground Level

Prioritization of health services imposed by the top management is crucial for the success of any health service, including adolescent-friendly health services. Nearly all respondents cited that adolescent health was often sidelined by higher authorities (state health department and Ministry of Health) with preference given towards non-communicable or infectious diseases. A 48-year-old female family medicine specialist stated the following:

“We still could not see the importance of adolescent health services. We don’t see adolescents as a group of people who needed care. Secondly, we focus too much on dengue fever, non-communicable diseases, diabetes, hypertension and heart problems. These are the problems that received more emphasis than adolescent health problems.”

(F1)

Respondents also believed, should the higher authorities give equal priority and emphasis on adolescent health services by consistently monitoring its progress, these services can be as successful as other scopes of health services. A 47-year-old female family medicine specialist said,

“I think more emphasis on adolescent health should be given by the higher authority … Through the audits (monitoring) done by higher authority, we will be able to pinpoint our weaknesses in the implementation process. From the audit findings, we can improve our services quality. If there is no audit or supervision, any program (health services) will fade away in time.”

(F3)

### 3.4. Theme 2: Organizational Supports to Advocate Adolescent-Friendly Health Services

Another important theme extracted was organizational support to advocate adolescent-friendly health services, derived from capacity building and financial aid, which acted as catalysts in the successful implementation of adolescent-friendly health services.

#### 3.4.1. Capacity Building to Ensure Competency of Healthcare Providers in Providing Adolescent-Friendly Health Services

The majority of respondents argued that training is necessary for staff competency in managing adolescent health problems. Training will assist staff to enhance their competency and knowledge on the methods of interacting and engaging with adolescents, and would eventually lead to better detection of adolescent health problems. A 50-year-old female public health physician made the following comment:

“We know that adolescents are not easy to be approached … So, if there is trained staff (on adolescent health management) and dedicated team in each clinic, surely adolescents will continue coming to the clinic. If staffs are not well-trained, they will not know the right way to engage with adolescents. Some staff even talked to adolescents in rude manner. This is very unwelcoming for them. Certainly adolescents will not come to this clinic again.”

(P1)

At the same time, respondents also shared their concern about the lack of training sessions on adolescent health services. The adolescent health training was conducted only once or twice per year, regardless at state or district level. A 50-year-old male family medicine specialist said,

“As for training (on adolescent health services), it is inadequate. Staffs from my clinic were only trained once at the district level. And at state level, training is only held once a year.”

(F5)

#### 3.4.2. Financial Aid to Sustain the Implementation of Adolescent-Friendly Health Services

Nearly all respondents were in agreement that it is important to have adequate budget to finance the establishment of adolescent-friendly clinics and adolescent health promotional programs. A 42-year-old male assistant medical officer stated the following:

“I think budget should be provided from higher authority. This is because as for now, adolescent health program does not have special budget allocation. If we were to organize adolescent health promotional activities especially outside of our clinic, we really need enough budget allocation.”

(A4)

Most healthcare professionals insisted on adequate monetary aid to finance adolescent health training sessions, refurbishment of clinic so that it will be more welcoming to adolescents, and also the provision of information, education, and communication (IEC) materials at clinics. As a 50-year-old male family medicine specialist highlighted,

“We need support which include financial support. To organize seminar, promotional activities and training sessions, we need adequate budget. If budget is enough, then it is easy for us to do any program. We need special budget to redecorate our clinic and to add more IEC materials (to suit adolescents’ need).”

(F5)

### 3.5. Theme 3: Appropriate Clinic Settings for Adolescent-Friendly Health Services

Appropriate clinic settings for adolescent-friendly health services refers to the arrangement of adequate privacy for adolescents and having a dedicated team for adolescent health services at clinic and implementing the family-doctor concept (FDC) at healthcare facilities.

#### 3.5.1. Providing Adequate Privacy for Adolescents at Clinics

All respondents concurred that adolescents prefer a private room for their consultation with healthcare providers as it provides convenience and confidence to share their sensitive problems. A 47-year-old male assistant medical officer made the following comment:

“If we have private room for adolescents, there will be no disturbance from other patients. When we have this kind of privacy, we can do consultation with teenagers in more detailed and focused way. Teenagers will be more confident (to share their secret) if only both of us (healthcare provider and teenager) are in the room. Otherwise, if other patients share the same room, teenager will not openly share their problems.”

(A3)

#### 3.5.2. Allocating a Dedicated Team for Adolescent Health Services at Clinics

In order to ensure good flow of adolescent case management at clinic level, the majority of respondents believed that it is imperative to have a dedicated team for adolescent health services at clinics. This dedicated team will be responsible for adolescent case registration, referral, follow-up, and data management. Most importantly, team members will become subject matter experts on adolescent health at clinic level, thus providing better screening and intervention for adolescent clients. As a 32-year old female medical officer commented,

“By having a special team (for adolescent health services), patient’s flow of management from the point of registration until consultation session will be more smooth. Adolescents do not have to wait so long to see doctor, and this is more convenient for them. Besides, this team will manage adolescents’ appointment and will facilitate follow-up session with them. This will surely attract more adolescent clients to the clinic.”

(M5)

#### 3.5.3. Implementing Family-Doctor Concept (FDC) at Healthcare Facilities

As adolescents are so concerned with the confidentiality of matters discussed with healthcare providers, the majority of respondents cited the importance of the family-doctor concept (FDC) at the clinics. Implementing family-doctor concept at each clinic would better manage adolescents since each staff member is familiar with their adolescent clients, including their family and social background. A 50-year-old female public health physician stated the following:

“In clinic with FDC, staffs work by zone, and they personally know well all clients in their respective zone, regardless of children, adolescents, adults or elderly clients. Thus, it is much easier for us to tackle their problems since we know so much about them and their family members. Personalized care is better with FDC system. So logically, FDC would help our health services to be more adolescent-friendly.”

(P1)

### 3.6. Theme 4: External Supports for Adolescent Health Promotional Activities

Among the key components of external supports for adolescent health promotional activities were inter-agencies collaboration and community involvement. Involvement of other agencies and community members helped to disseminate information on adolescent health services to community, particularly adolescents, and directly increase the uptake of services among adolescent clients.

#### 3.6.1. Inter-Agencies Collaboration in Promoting Adolescent Health Services

Many respondents, particularly staffs who were actively involved in the promotional activities, insisted on the importance of having inter-agencies collaboration in facilitating healthcare facilities to promulgate adolescent-friendly health services to adolescents at large. By incorporating other agencies as stakeholders in the promotional activities, healthcare providers would receive additional manpower with technical expertise and also additional IEC materials to be used for health promotional purposes. A 33-year-old female medical officer commented as follows:

“Collaboration with them (other agencies) is important because we lack manpower. LPPKN (The National Population and Family Development Board) and ReHAK (Reproductive Health Association of Kelantan) can help us during promotional activities if we invite them. They have many modules and IEC materials (pertaining to adolescent health) which can be used during our promotional activities.”

(M2)

Furthermore, respondents highlighted that collaborating with other agencies, particularly with educational institutions, would facilitate healthcare providers to reach out to more adolescent clients. As a 44-year-old female nurse stated,

“Secondly, we have to collaborate with other agencies in doing health promotion, especially schools. Schools are very important since majority of the adolescents are school-goers. So we can use school as a platform for us to reach adolescents and inform them about the availability of adolescent health services at our clinic.”

(N3)

#### 3.6.2. Community Involvement in Adolescent Health Promotion

Many respondents claimed good rapport with community members and their direct involvement in promotional activities would assist healthcare facilities to disseminate adolescent-friendly health services to local youths. Community members, particularly parents who are aware about the availability of adolescent-friendly health services at their local clinics, would exert their power to encourage their children to avail these health services at local clinics. As a 31-year-old female medical officer commented,

“We ought to have good rapport with the community, especially with the parents so that they will encourage their teenage children to join our program (adolescent-friendly health services).”

(M1)

## 4. Discussion

Based on our previous study, the coverage of clinics providing adolescent-friendly health services in Kelantan state was quite low, which was 35.3% [19], substantially lower than the coverage of adolescent-friendly health services in our neighboring country, Indonesia. The proportion of adolescent-friendly health facilities in Indonesia was 52.65% in 2017, which met their national target of 35% [31]. From the qualitative findings, the scarcity of adolescent-friendly clinics could be attributed to the lack of emphasis and training from higher authority on adolescent health services, relative to other scopes of health services involving acute illnesses and infectious diseases which require urgent attention. A similar finding was also reported in an Ecuadorian study, where adulthood health services were more privileged over adolescent health services [20].

Our quantitative results of the current study revealed the number of healthcare providers, daily patient attendance, types of clinic, and availability of family medicine specialist at clinics were not significantly related to the adolescent-friendliness level of clinics. It was discovered that commitment from healthcare provider played a more vital role in determining the success of adolescent-friendly clinic. As demonstrated in another study, implementing adolescent-friendly health services required extra effort and commitment from healthcare providers in the form of extra working hours or even money as the services were not fully institutionalized [20].

Besides that, financial support and FDC system may play an important role in the enhancement of the service provision, similar to studies done in Ecuador and Ethiopia. Researchers in these two countries echoed that financial aid and integration of family-doctor concept at clinic are key components for adolescent-friendly health services [20,21]. In any type of clinic, our qualitative respondents emphasized the importance of having adequate budget to finance the establishment of adolescent-friendly clinics and their programs. It is already demonstrated in Estonia that the provision of sustainable national funding to adolescent health services allowed youth-friendly clinics to focus on long-term planning, quality improvement, and sustainable scale-up of the services. In return, the economic growth in Estonia is positively influenced by the health status of the adolescent population due to the sustainability of youth clinic funding [32].

Besides financial support, the role of FDC is also paramount in facilitating the provision of adolescent-friendly health services. In Malaysia, the Ministry of Health started the FDC in 2013 to invigorate primary healthcare service in Malaysia and to achieve the target of “One Family One Doctor”. This is a concept in which a doctor in a clinic will be assigned to a dedicated family to take the responsibility of care from womb to tomb. Being followed up and treated by the same healthcare providers will create a good patient-doctor relationship, provide the longitudinal care which can avert diseases, and enhance the quality and continuity of care and client’s compliance towards the treatment and management provided by the clinics [33]. Therefore, regardless of clinic workload, our qualitative respondents working at clinics with an FDC system reported to have better understanding about adolescent clients, including their social and family background, hence ensuring easier and more efficient case management. Paradoxically, in a Canadian study, it was reported that the FDC system is not compatible with adolescent-friendly clinics, as adolescents fear that the family doctor might divulge their parents about their secrets and highly sensitive problems [34].

The result of our study showed that highly trained healthcare providers at clinic was significantly related to the adolescent-friendliness level of clinic because capacity building among staff will ensure our healthcare providers are knowledgeable and competent enough to approach adolescents and respond to their needs, as highlighted in our qualitative exploration. Engaging with adolescents to tackle their health problems requires training of various levels of healthcare personnel and strategies to build capacities that are immediately needed. This could involve promotive, preventive, and curative components in the existing training or newer initiatives for adolescent health management [35]. In line with our study findings, Motuma, Syre, Egata, and Kenay [21] reported in their study that competency and knowledge of providers will play a vital role in the kind of information youths will obtain and indirectly determine the health outcome of adolescents. Apart from that, a systematic review revealed that it is important for young clients to feel included and autonomous during health communication. Thus, healthcare providers would require specific skills through special training to balance these needs with the parental influence [36]. However, despite the importance of highly trained healthcare providers in providing adolescent-friendly health services, our qualitative respondents highlighted the lack of training in the field of adolescent health management. Our qualitative finding is congruent with the data from the Ministry of Health, Malaysia which reported only 4.62% of the healthcare budget was dedicated for training activities. Besides, training programs in adolescent health were relatively lower than in other divisions, such as the health education division and the food safety division [37].

In this study, availability of private room or space for adolescent counseling session was significantly related to adolescent-friendliness level, and it was justified qualitatively that the private areas for adolescents are important to build confidence for them to openly share their problems and eventually lead to effective and fruitful consultation sessions. Our study finding is congruent to a Swedish study in which their healthcare providers also cited the space of the clinics should warrant spatial confidentiality during consultation in order to provide convenience for youths [22]. Moreover, spatial confidentiality would render emotional safety to adolescents during healthcare visits, and this will promote open and engaging communication and lead to fruitful discussions [36].

Our quantitative results showed dedicated team in charge of adolescent health services was a significant predictor for adolescent-friendliness level of clinics. Our healthcare providers explained that a dedicated team will ensure more efficient management of adolescent cases; and more systematic management of data and appointment at clinic level. Similarly, in Sweden, having a dedicated team is an important element to ensure a comprehensive response to the multiple youth health needs and to ensure staffs feel supported in regards to their workload [22].

Quantitatively, the present study also indicated promotional activities to promote adolescent health services to adolescents and community was significantly related to adolescent-friendliness level of clinics. Qualitatively, healthcare providers explained that community involvement and inter-agencies collaboration, particularly with educational institutions, are important factors required to help disseminate information on adolescent-friendly health services to adolescents. These stakeholders can provide not only technical expertise, but also financial support and manpower for clinics to do promotional activities to the community, particularly adolescents, which eventually enhance the effectiveness of health promotion and increase service utilization. As reported by other studies, promotion of health services is important because these services are only accessible for adolescents who know about the clinics [20,22]. Besides, acknowledgement of the services from the community and close support from organizations with extensive knowledge on youth health will encourage services improvement; and good collaboration with schools is important because it helps to promote youth clinics among potential users [20,22].

Among the limitations of our study is the limited account of the adolescents’ perceptions regarding the facilitating factors for provision of adolescent-friendly health services. Our previous study has demonstrated that adolescent clients were more satisfied in utilizing services in adolescent-friendly clinics in relation to conventional clinics [19]. Therefore, it would be beneficial to obtain adolescents’ input pertaining to their demands and expectations on ideal setting of adolescent-friendly clinics to maximize the utilization of services. However, following the best practice criteria for adolescent-friendly health services as outlined by the Ministry of Health, Malaysia [7], this present study will complement the findings of our previous assessment study on coverage of adolescent-friendly health facilities to guide policymakers and healthcare providers to strengthen the current adolescent health services in Malaysian primary healthcare facilities [19]. Apart from the previously mentioned limitation, in doing qualitative study there will be limited generalization of qualitative findings to Malaysian level, as these findings can only be inferred to states in Peninsular Malaysia due to high index of similarity in geographical and socio-demographic features. Malaysian states in Borneo part may have different views on the facilitating factors for provision of adolescent health services, such as in term of accessibility to health services, as the majority of people with the highest inaccessibility to a health service reside in Malaysian Borneo [38,39]. To better represent Malaysian setting and better echo the needs of both providers and clients, it is recommended that future qualitative studies should be done among both adolescent clients and healthcare providers in other Malaysian states, particularly states with different geographical and socio-demographic features, such as Sabah and Sarawak located in the Borneo island of East Malaysia.

## 5. Conclusions

In conclusion, adolescent-friendly health services in the Malaysian setting have great potential to be expanded in the near future should the government give more priority to adolescent health services and needs. Our present study demonstrates that mixed-methods study design can be utilized for a better understanding on the facilitating factors towards providing adolescent-friendly health services in the Malaysian setting, particularly the peninsular part of Malaysia. The Ministry of Health, Malaysia is recommended to respond towards the required facilitating factors by providing more training for healthcare providers and a dedicated area for adolescents in clinics; setting up a dedicated adolescent health team in the clinic; strengthening promotional activities to adolescents and community; allocating adequate budget to adolescent health services; and expanding family-doctor concept to all primary healthcare facilities in order to further improve the quality of adolescent health services in Malaysia.

## Figures and Tables

**Table 1:** Factors related with clinic assessment score among clinics in Kelantan by simple and multiple linear regression (*n* = 85).

Variables	Simple Linear Regression		Multiple Linear Regression		
	Crude b (95% CI)	*p*-Value	Adjusted b (95% CI)	*t*-Stat	*p*-Value
Number of healthcare providers	0.37 (0.20,0.54)	<0.001	0.06 (−0.23,0.35)	0.40	0.690
Daily patients’ attendance	0.04 (0.02,0.05)	<0.001	−0.007 (−0.03,0.02)	−0.53	0.598
Type of clinic					
Universal	1.00		1.00		
Intermediate	7.35 (1.45,13.26)	0.015	0.29 (−4.26,4.86)	0.13	0.896
Advanced	19.51 (12.13,26.89)	<0.001	0.93 (−8.37,10.23)	0.19	0.843
Availability of family medicine specialist at clinic					
No	1.00		1.00		
Yes	13.44 (7.43,19.45)	<0.001	−0.50 (−6.67,5.66)	−0.16	0.871
Availability of highly trained healthcare provider in adolescent health management					
No	1.00		1.00		
Yes	14.98 (9.99,19.96)	<0.001	5.64 (1.54,9.73)	2.68	0.007
Availability of private room or space for counseling session					
No	1.00		1.00		
Yes	15.65 (9.89,21.40)	<0.001	5.66 (1.45,9.87)	2.68	0.009
Availability of dedicated team in charge of adolescent health services.					
No	1.00		1.00		
Yes	19.23 (14.65,23.82)	<0.001	9.07 (4.68,13.47)	4.12	<0.001
Availability of promotional activities to promote adolescent health services to adolescents and community					
No	1.00		1.00		
Yes	21.23 (16.65,25.82)	<0.001	13.11 (8.46,17.75)	5.62	<0.001

R^2^ = 73.1%; stepwise/backward/forward multiple linear regression applied; model assumptions are fulfilled; no interactions among independent variables; no multicollinearity detected.

**Table 2:** Attributes of qualitative participants (*n* = 23).

Variables	Frequency (%)
Age (year) *	46 (16)
Working experience (year) *	20 (14)
Gender	
Male	6 (26.1)
Female	17 (73.9)
Education level	
Diploma level	8 (34.8)
Bachelor’s degree	7 (30.4)
Master’s degree	8 (34.8)
Job title	
Public health physician	3 (13.1)
Family medicine specialist	5 (21.7)
Medical officer	6 (26.1)
Assistant medical officer	4 (17.4)
Nurse	5 (21.7)

* Median (IQR).

**Table 3:** Categories and final themes on the facilitating factors for adolescent-friendly health services.

Categories	Themes
Self-commitment of healthcare providers to provide adolescent-friendly health services. Prioritization by top managers towards the implementation of adolescent-friendly health services at ground level.	Theme 1: Healthcare providers’ commitment and prioritization towards adolescent-friendly health services.
Capacity building to ensure competency of healthcare providers in providing adolescent-friendly health services. Financial aid to sustain the implementation of adolescent-friendly health services.	Theme 2: Organizational supports to advocate adolescent-friendly health services.
Providing adequate privacy at clinics for adolescents. Allocating a dedicated team for adolescent health services at clinic. Implementing the family-doctor concept (FDC) at healthcare facilities.	Theme 3: Appropriate clinic settings for adolescent-friendly health services.
Inter-agencies collaboration in promoting adolescent health services. Community involvement in adolescent health promotion.	Theme 4: External supports for adolescent health promotional activities.
